# Recurrent Olfactory Neuroblastoma Treated With Cetuximab and Sunitinib: A Case Report

**DOI:** 10.1097/MD.0000000000003536

**Published:** 2016-05-06

**Authors:** Lizhi Wang, Yan Ding, Lai Wei, Dewei Zhao, Ruoyu Wang, Yuewei Zhang, Xuesong Gu, Zhiqiang Wang

**Affiliations:** From the Department of Otolaryngology (LW, LW, ZW); The Institute for Translational Medicine, Affiliated Zhongshan Hospital of Dalian University, Dalian, China (YD, DZ, RW, YZ); Genomic Future, Inc, Lexington, MA (YD); and Beth Israel Deaconess Medical Center, Harvard Medical School, Boston, MA (XG).

## Abstract

Olfactory neuroblastoma (ONB) is a rare cancer originating in the olfactory epithelium of the nasal vault. The recurrence rate of ONB is high, as the standard treatment of surgery followed by radiotherapy and/or chemotherapy is usually unsuccessful. The use of targeted therapy based on individual genomic variations after cancer relapse has not been reported. Here, we present the case of a 44-year-old man who was diagnosed with recurrent ONB and treated with a regimen developed using whole exome sequencing. Potential targets were first identified and then matched to appropriate drugs. Gene mutations in the genes encoding EGFR, FGFR2, KDR, and RET were discovered in the patient's tumor tissue by whole exome sequencing and the patient was treated with a combination of the targeted drugs cetuximab and sunitinib. Five days after treatment, enhancement magnetic resonance imaging showed a 65% reduction in tumor size, and the Visual analog scale headache scores went down to 2/10 from 10/10. Repeat imaging at 1 month showed a complete response.

This study represents the first demonstration of an effective personalized treatment of ONB by targeted drugs, and sheds light on how precision medicine can be used to treat recurrent ONB that fails to respond to routine tumor resection, radiotherapy, and/or chemotherapy.

## INTRODUCTION

Olfactory neuroblastoma (ONB) is also called esthesioneuroblastoma, which is a rare and slow-growing malignant tumor arising in the olfactory epithelium located in the upper part of nasal cavities, the ectopic sphenoclival part,^1^ or the sphenoid sinus.^2^ ONB comprises 3% to 5% of nasal cancers with an incidence of 1 per 2.5 million.^3^ The etiology of ONB is unclear. The recurrence rate and mortality of ONB remain high. Patients commonly complain of epistaxis, nasal obstruction, and olfactory and ophthalmic disturbances, as well as craniofacial pain. Some patients present with florid Cushing syndrome that is secondary to ONB,^4^ or paraneoplastic syndromes and ectopic adrenocorticotropic hormone syndrome.^5^ A diagnosis of ONB may be established by histopathology and confirmed by immunohistochemistry. The incidence of cervical lymph node metastasis in ONB is variable, and few reports have been published concerning retropharyngeal lymph node metastasis from ONB.^6^ There is no defined treatment protocol for this disease. Surgical resection combined with postoperative radiotherapy has been described as the standard of care for primary site tumor.^7^ However, the optimal treatment continues to be controversial because of the rarity of the disease. Targeted therapy with either small molecule or monoclonal antibody drugs in guide of genomics has not been reported. In the present case, a patient diagnosed with ONB had gone through 3 rounds of transnasal endoscopic surgery followed by radiotherapy (60Gy) and chemotherapy, but presented with a recurrence of ONB 5 months after this standard treatment regimen. Identification of genomic variations in the tumor tissue made via whole exome sequencing led to the development of a targeted therapy regimen using a combination of cetuximab and sunitinib. The clinical outcome of this new approach to the treatment of ONB is reported.

### Case Report

**FIGURE 1:**
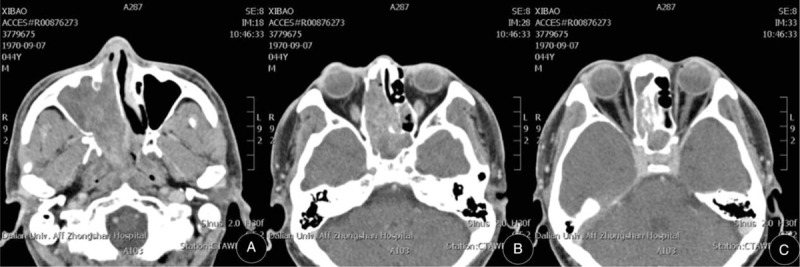
Paranasal sinus computed tomography scan shows paranasal sinus involvement (A), skull base erosion (B), orbit infiltration and intracranial involvement (C).

**FIGURE 2:**
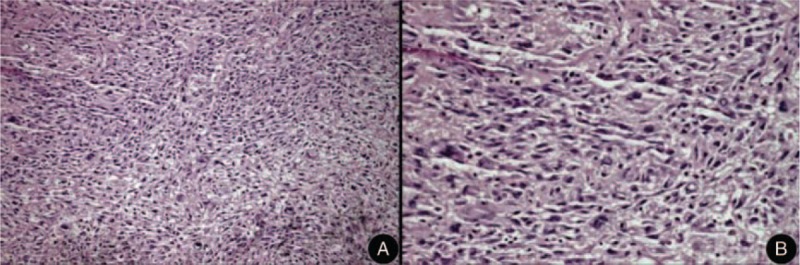
Microscopically, sheets or discrete nests or lobules of small round cells slightly larger than lymphocytes are present, which are often compartmentalized into nodules by thin fibrous septa. (A) H&E ×100. (B) H&E ×400.

**FIGURE 3:**
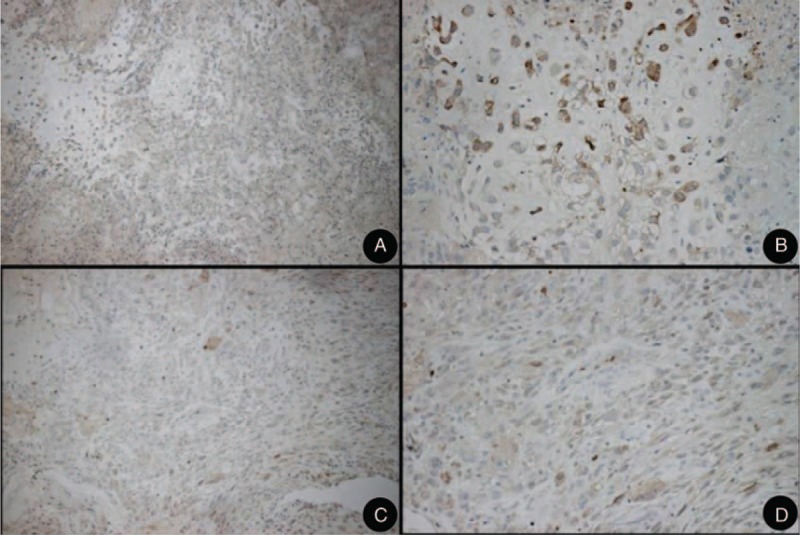
Immunohistochemically, olfactory neuroblastomas stain for Neuron Specific Enolase (NSE) (A, B). The supporting or sustentacular cells tested positive for S-100 protein (C, D).

A 44-year-old male was diagnosed with ONB and underwent an operation on August 2014. He complained of nasal obstruction, rhinorrhea, and intermittent epistaxis starting 8 months previous to this, and of cacosmia for 1 day. Dark red neoplasm located in the patient’ right nasal cavity was observed. Computed tomography scan further clearly showed the invasion of multiple structures including anterior skull base, orbit, frontal sinus, ethmoid sinus, maxillary sinus, sphenoid sinus, and nasal septum (Figure 1). Pathological results showed that the tumor cells were ONB (Figure 2 and Figure 3).

The patient refused orbital exenteration, but accepted endoscope-assisted tumor radical excision. After the operation, he received radiotherapy of 60 Gy in fractions of 2 Gy and 3 courses of chemotherapy, including ifosfamide (IV, once a day, 3 g daily for 5 days), cisplatin (IV, once a day, 45 g daily for 3 days), and etoposide (IV, once a day, 0.11 g daily for 5 days), but refused continued chemotherapy because of serious side effects including arthralgia, serious nausea and vomiting, oral ulcer, and hair loss.

**FIGURE 4:**
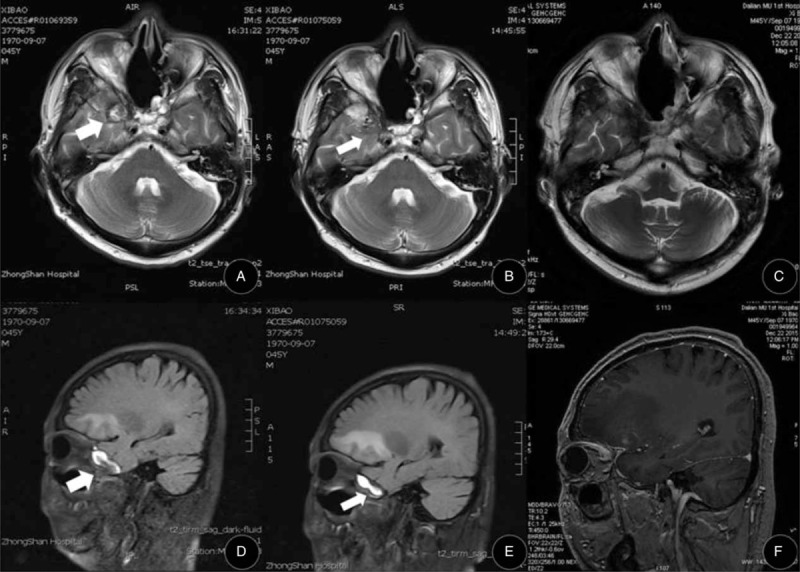
(A and D) Enhancement cerebral magnetic resonance imaging (MRI) showing the right temporal lobe space-occupying lesion (white arrows): (A) Horizontal T2 imaging; (D) Sagittal T2 imaging (size: 15.8 × 13.6 × 14.3 mm). (B and E) Five days after the cetuximab and sunitinib treatment, enhancement cerebral MRI shows the right temporal lobe space-occupying lesion (white arrows) is reduced in size with blurred boundary to peripheral tissue: (B) Horizontal T2 imaging; (E) Sagittal T2 imaging (size: 12.6 × 9.1 × 9.3 mm). (C and F) One month after treatment, cerebral MRI shows that the right temporal lobe space-occupying lesion has completely disappeared: (C) Horizontal FSE imaging; (F) Sagittal M3D/BRAVO imaging.

Five months after postoperative treatment was discontinued, the patient presented with complaints of headache and abulging right eye with decreased vision for 1 day. Examination showed that he had loss of vision, eyeball fixation, and blephroptosis of the right eye. Visual analog scale (VAS) scores of headache pain were 10/10. Cerebral enhancement magnetic resonance imaging (MRI) distinctly showed that there was a lesion occupying the right temporal lobe space (size: 15.8 × 13.6 × 14.3 mm). Tumors recurred and affected the nearby extraocular muscles and the optic nerve (Figure 4A, D).

Given the failure of standard radiotherapy and chemotherapy after tumor resection, we decided to explore the options for targeted therapy for this patient. Whole exome sequencing (WES) was employed to detect the target gene mutations from patient tumor/normal tissue pairs on the Illumina NextSeq500 sequencing platform and using a TruSeq Rapid Capture Exome Kit for library construction. The WES data were then analyzed using OncoDecoder^TM^ (Genomic Future, Inc). The significantly mutated cancer-related genes that were identified in tumor tissue included *EGFR*, *KDR*, *FGFR2*, and *RET* (Table 1).

**TABLE 1:**
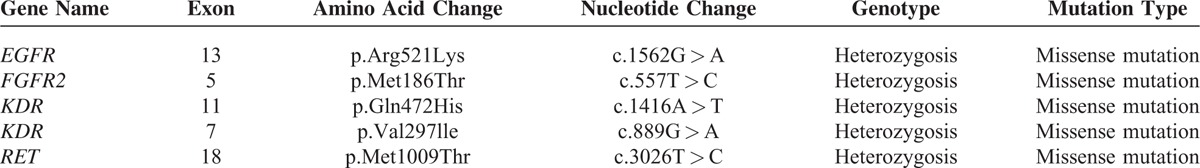
The Significantly Mutated Genes From the Tumor Tissue of the Patient Detected By Whole Exome Sequencing

Specifically, we found a missense mutation p.Arg521Lys in exon13 of *EGFR*, missense mutations p.Gln472His (exon11) and p.Val297lle (exon7) in *KDR*, a missense mutation p.Met186Thr in exon5 of *FGFR2*, and a missense mutation p.Met1009Thr in exon 18 of *RET*. Additionally, the *KRAS* gene was identified as wild type. Quantitative real-time polymerase chain reaction confirmed the overexpression of *EGFR* and *KDR* genes in the tumor tissue.

Based on these findings, we carefully screened the currently available targeted drugs that act on the list of mutated cancer-related genes and that are used in head and neck cancers. We determined a treatment regimen of a combination of cetuximab and sunitinib, wherein cetuximab is a targeted drug for *EGFR* and sunitinib for *KDR*, *FGFR2*, and *RET*. The ethics committee of the Affiliated Zhongshan Hospital of Dalian University approved the study. The patient received concurrent cetuximab (IV, once per week, 300 mg each time for 4 weeks, initial dose is 600 mg) and sunitinib (PO, once a day, 50 mg daily for 4 weeks) treatment. Five days later, enhancement MRI revealed right that the lesion occupying the right temporal lobe space had shrunk to a small hypointense area (size: 12.6 × 9.1 × 9.3 mm). VAS scores of headache pain were down to 2/10, although there was still loss of vision, eyeball fixation, and blephroptosis (Figure 4 B, E). Red Erythra without itch appeared 10 days after cetuximab was administered to the patient and disappeared completely in 2 weeks. The patient was also found in slightly feeble condition, which was confirmed by low potassium in serum, and symptom was disappeared after potassium chloride intake. Oral ulcer healed but not hair loss because of early chemotherapy. After he was given the third dosage of cetuximab, MRI showed that the right temporal lobe space lesions had completely disappeared (Figure 4C, F). As we prepared the final draft of our manuscript, the patient remains in stable state, and it has been >4 months since he was clinically cured of ONB.

## DISCUSSION

The rate of neck recurrence in ONB is close to 15%, among those patients who developed regional metastases, the mortality is 60%, and the overall mortality is 32%.^8^ Determining the optimal treatment scenario for recurrent ONB is challenging. A combination of endoscopic/open surgery, radiotherapy, and/or chemotherapy has been considered as standard care for ONB. Concurrent treatment with neoadjuvant and adjuvant chemotherapy has also been described,^9^ but the impacts on clinical outcomes remain unknown. Unfortunately, the traditional treatment regimens often fail for recurrent and metastatic ONB cases. Moreover, some patients may refuse to take the standard protocols because of their intolerance of radiation and/or chemotherapy, like the patient in our case. Most recently, genome-based precision medicine has drawn a great deal of attention from oncologists. Several studies have shown that the use of targeted medicines can result in either a complete response (CR) or a significant improvement in the quality of life of patients with a variety of cancers.^10–12^ It is widely accepted that one of the biggest advantages of targeted drugs is their high specificity and low toxicity. The targeted drugs used in this treatment are examples of the 2 basic types of targeted drugs. Sunitinib is an example of small molecule-targeted drug,^13^ it exerts its antitumor effects by inhibition of a wide spectrum of receptor tyrosine kinases, including vascular endothelial growth factor (VEGF) receptors (VEGFR2/KDR), fibroblast growth factor receptors (FGFR1 and FGFR2), platelet-derived growth factor receptors (PDGFRA and PDGFRB), fetal liver tyrosine kinase receptor (FLT1, FLT3, and FLT4), RET, and c-Kit.^14–16^ Sunitinib was approved by Food and Drug Administration (FDA) in 2011 for treatment of metastatic renal cell carcinoma, metastatic or unresectable gastrointestinal stromal tumors (GIST), and unresectable or metastatic pancreatic neuroendocrine tumors. Cetuximab^17^ is an example of the class of biological conjugates. It is a monoclonal antibody to the epidermal growth factor receptor (EGFR). Cetuximab has been approved for treatment of head and neck cancers by US FDA in 2004 and has been used to treat squamous carcinomas of the head and neck and colorectal cancers (CRCs) with wild-type KRAS. In a previous study by Preusser et al^18^ sunitinib showed significant improvement of clinical symptoms and disease stabilization to an ONB patient, who had positive staining of PDGFRB in the tumor tissue specimen by immunohistochemistry. However, to the best of our knowledge, personalized treatment of ONB with targeted drugs based on the patient's genomic variations has not been reported.

To explore the opportunity and potential benefit of targeted therapy for advanced and recurrent ONB, we sequenced the whole exomes of the tumor/normal tissues of the patient in our genetic test laboratory, and identified several significantly mutated cancer genes including *EGFR*, *VEGFR2/KDR*, *FGFR2*, and *RET* (Table 1). Interestingly, the R521K polymorphism of the *EGFR* gene was associated with a longer progression-free survival in CRC patients treated with cetuximab.^19^ Therefore, it is perhaps not surprising to see that the patient showed a striking response to a cetuximab plus sunitinib treatment regimen (Figure 4). One month after the target therapy, the patient showed a CR. As we prepared the final draft of our manuscript, the patient remains in stable state. Follow-up care has been established for the patient and an update of clinical outcomes is warranted. As ONB is a rare disease, collaborative efforts for a cohort study is desirable to further prove the effectiveness of targeted therapy for ONB.

## CONCLUSIONS

Surgery followed by radiation and chemotherapy is the current standard of care for ONB and fails frequently with a high risk of morbidity and severe side effects. Genome-based targeted therapy for recurrent and late-stage ONB is certainly an option in terms of its promising clinical response, and hence deserves further investigation in a prospective clinical trial.
